# PREVALENCE OF POSSIBLE SARCOPENIA AND ASSOCIATED FACTORS AMONG OLDER HOMELESS ADULTS IN HOME FOR DESTITUTE

**DOI:** 10.1093/geroni/igae098.4052

**Published:** 2024-12-31

**Authors:** Phatcharaphon Whaikid, Noppawan Piaseu, Wasana Srisuk

**Affiliations:** Mahidol University, Bangkok, Krung Thep, Thailand; Mahidol University, Bangkok, Nakhon Pathom, Thailand; Mahidol University, Bangkok, Nakhon Pathom, Thailand

## Abstract

Sarcopenia is a significant problem leading to increased morbidity and mortality risk, particularly in older homeless adults in home for destitute as they are vulnerable and are experiencing premature degeneration. The Asian Working Group for Sarcopenia (AWGS) 2019 introduces possible sarcopenia for early prevention of sarcopenia. Therefore, this study aimed to describe the prevalence and risk factors of possible sarcopenia among older homeless adults. This cross-sectional study included 163 older homeless adults aged 50 and above in homes for destitute in Thailand. The participants completed a questionnaire on personal and health-related factors, and assessments according to AWGS 2019 criteria. Data were analyzed using descriptive statistics and multinomial logistic regression. Results revealed that the prevalence of case finding, and possible sarcopenia was 74.9% and 71.2%, respectively. Among participants aged 50-59, the prevalence rates were 60.8% and 55.1%, while those aged 60 years and over showed a significantly higher prevalence of 90.8% and 89.5%, respectively. Multinomial logistic regression analysis revealed four risk factors significantly associated with possible sarcopenia, including age, gender, BMI, and number of comorbidities (odds ratio (OR) = 1.253, 95% confidence interval (CI) = 1.117-1.405, OR =.222, 95% CI =.056-.874, OR =.584, 95% CI =.458-.745, and OR =.308, 95% CI =.109-.868, respectively). This study confirmed that possible sarcopenia can begin as early as age 50, aligning with indications of premature degeneration in this population. Therefore, it is recommended to start screening earlier than usual to ensure proper management and delay the progression of sarcopenia.

